# Multicentric Round Cell Neoplasia with Plasmacytic Differentiation in a Cat with Systemic Progression: Multimodal Imaging and Treatment Response

**DOI:** 10.3390/ani16071089

**Published:** 2026-04-02

**Authors:** Jaewon Kim, Inseong Jeong, Chul Park, Younghwan Kim, Kidong Eom, Jaehwan Kim

**Affiliations:** 1 Department of Veterinary Medical Imaging, College of Veterinary Medicine, Konkuk University, Seoul 05029, Republic of Korea; solid0209@konkuk.ac.kr (J.K.);; 2 Royal Animal Medical Center, Seoul 02140, Republic of Korea

**Keywords:** round cell neoplasia, plasmacytic differentiation, multimodal imaging, radiation therapy, cat

## Abstract

Plasma cell neoplasia is uncommon in cats, and multicentric nodal-predominant involvement has not been well characterized. This report describes a multicentric round cell neoplasia with plasmacytic differentiation in a 14-year-old Domestic Shorthair cat, emphasizing multimodal imaging features and treatment response. Contrast-enhanced computed tomography and magnetic resonance imaging were performed for staging and longitudinal assessment. Cytomorphology supported plasmacytic differentiation, and flow cytometry did not demonstrate an immunophenotype consistent with conventional B- or T-cell lymphoma. Because histopathology, immunohistochemistry, bone marrow evaluation, and assessment for monoclonal gammopathy were not performed, definitive classification was not possible; however, cytomorphology supported plasmacytic differentiation, with plasma cell neoplasia remaining an important diagnostic consideration. A hypofractionated radiotherapy protocol (36 Gy in six fractions) combined with systemic chemotherapy was administered. Serial imaging demonstrated complete radiologic resolution of the irradiated mass, whereas non-irradiated presumed nodal lesions progressed and an extradural spinal lesion subsequently developed. These findings highlight the capacity of round cell neoplasia with plasmacytic differentiation to mimic lymphoma on imaging and illustrate the dissociation between effective local control and ongoing systemic progression.

## Simple Summary

Plasma cell neoplasms are uncommon in cats, and their imaging appearance and treatment response are not well documented, particularly when disease is distributed predominantly at lymph node sites. This report describes an older cat with multiple masses in the abdomen and chest that were considered most consistent with enlarged lymph nodes on imaging. Ultrasound, computed tomography, and magnetic resonance imaging were used to determine disease extent and monitor treatment response over time. Examination of cells collected from the largest abdominal mass supported plasmacytic differentiation and raised plasma cell neoplasia as an important diagnostic consideration in addition to the more common diagnosis of lymphoma. The cat was treated with radiation therapy directed at the largest mass together with chemotherapy. Follow-up imaging showed a rapid and marked decrease in the size of the treated mass, which later became undetectable on imaging. In contrast, other presumed nodal lesions progressed, and a new mass later developed near the spinal cord. This case shows that round cell neoplasia with plasmacytic differentiation may closely resemble lymphoma on imaging studies, and that repeated imaging is valuable for evaluating treatment response and disease progression.

## 1. Introduction

Myeloma-related disorders (MRDs) arise from the neoplastic transformation of plasma cells or immunoglobulin-secreting B lymphocyte precursors and encompass a heterogeneous spectrum of plasma cell neoplasms. In cats, MRDs are classified as multiple myeloma (MM), cutaneous extramedullary plasmacytoma (CEMP), non-cutaneous extramedullary plasmacytoma (NCEMP), solitary plasmacytoma of bone (SPB), IgM macroglobulinaemia, immunoglobulin-secreting lymphoma, immunoglobulin-secreting leukaemia, and plasma cell leukaemia [1,2]. These disorders are rare in feline patients, accounting for approximately 0.003–0.1% of all reported malignancies in large case series [3]. In comparison, MRDs represent less than 1% of all tumours in dogs [4] and approximately 1–2% of all neoplasms in humans [5].

Among the spectrum of MRDs, extramedullary plasmacytoma (EMP) is defined as a localized proliferation of neoplastic plasma cells occurring in soft tissues without primary evidence of bone marrow involvement [1]. In cats, non-cutaneous extramedullary plasmacytomas (NCEMPs) have been reported in a variety of anatomical locations, including the spleen [1,6], liver [1], intracerebral tissue [7], intraocular tissue [8], orbital tissue [1,9], respiratory tract [10,11,12], subcutaneous tissue [13], oral cavity [6,14], mesentery [15], stomach [16], and duodenum [17]. Although lymph node involvement has been reported in feline plasma cell neoplasia [1], a multicentric nodal-predominant presentation documented with serial cross-sectional imaging has not been well characterized.

The present report describes a multicentric round cell neoplasia with plasmacytic differentiation in a cat, with lesions distributed predominantly at expected lymph node stations. To the best of our knowledge, this is the first feline case to provide serial cross-sectional imaging documentation of this disease presentation, including objective assessment of local response after radiation therapy and concurrent progression at non-irradiated sites during systemic treatment.

## 2. Case Presentation

A 14-year-old castrated male Domestic Shorthair cat weighing 6.8 kg was referred for further evaluation of an incidentally detected intra-abdominal mass identified at a local veterinary clinic. The owner reported no gastrointestinal signs, including vomiting, diarrhea, anorexia, or weight loss, and no clinical abnormalities attributable to the mass had been recognized prior to referral.

Physical examination revealed no palpable peripheral lymphadenopathy, and abdominal palpation did not identify a clearly discernible focal mass. Vital parameters were within normal limits.

A complete blood count was within reference intervals. Serum biochemistry analysis revealed mild hyperproteinemia (total protein 8.5 g/dL; RI, 5.7–8.2 g/dL) characterized by hyperglobulinemia (5.7 g/dL; RI, 2.6–5.1 g/dL) and a borderline low albumin concentration (2.7 g/dL; RI, 2.7–3.9 g/dL), resulting in a decreased albumin-to-globulin ratio (0.49; RI, 0.6–1.1). Serum calcium concentration was within normal limits (10.4 mg/dL; RI, 9.0–11.6 mg/dL). Serum amylase activity was elevated (2713 U/L; RI, 433–1248 U/L), and serum amyloid A (SAA) concentration was markedly increased (94.5 µg/mL; RI, 0–5 µg/mL). Serum symmetric dimethylarginine (SDMA) concentration and coagulation parameters were within reference intervals. Urinalysis demonstrated hyposthenuria (urine specific gravity 1.008) without proteinuria, and Bence–Jones protein testing was negative.

Abdominal ultrasonography was performed using a high-resolution ultrasound system (Aplio i700; Canon Medical Systems, Tustin, CA, USA). Examination revealed multiple intra-abdominal masses at expected lymph node stations, interpreted as most consistent with marked enlargement of presumed abdominal lymph nodes. These masses were heterogeneously hyperechoic with mixed internal echotexture, and increased echogenicity of the adjacent mesenteric fat was observed.

**Figure 1:**
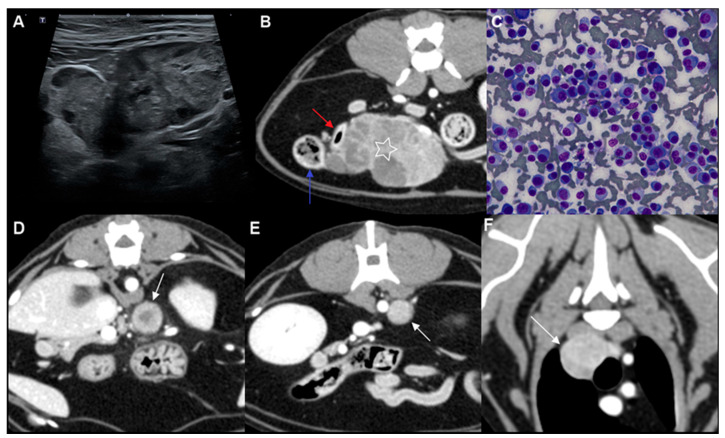
Multimodal imaging and cytologic features of the mass located adjacent to the ileocolic junction, with representative contrast-enhanced CT images of additional lesions at expected abdominal and thoracic lymph node stations. For all CT panels (**B**,**D**–**F**), the right side of each image corresponds to the patient’s left. (**A**) Abdominal ultrasound image demonstrating a heterogeneous hyperechoic mass with mixed internal echogenicity adjacent to the ileocolic junction. (**B**) Post-contrast CT image (axial plane; window level, 40; window width, 400) showing the contrast-enhancing mass (white star). The adjacent ileum (red arrow) and colon (blue arrow) are indicated. (**C**) Cytologic smear revealing a highly cellular population predominantly composed of round cells with plasmacytic differentiation, characterized by eccentrically positioned nuclei, coarse chromatin, and pale perinuclear clearing (perinuclear hof). Occasional binucleated and multinucleated cells are observed. (**D**) Post-contrast CT image (axial plane; window level, 40; window width, 400) demonstrating a contrast-enhancing lesion at the expected location of the splenic lymph node (white arrow). (**E**) Post-contrast CT image (axial plane; window level, 40; window width, 400) demonstrating a contrast-enhancing lesion at the expected location of the lumboaortic lymph node (white arrow). (**F**) Post-contrast CT image (axial plane; window level, 40; window width, 400) demonstrating a contrast-enhancing lesion at the expected location of a cranial mediastinal lymph node (white arrow).

The largest mass was identified near the ileocolic junction and was interpreted as most consistent with enlargement of the presumed colic lymph node (Figure 1A). This lesion measured 53 mm × 28 mm and demonstrated an irregular margin. A single additional nodule measuring approximately 8–9 mm in diameter was identified in close proximity to this mass. A rounded mass adjacent to the splenic vein, measuring 13.3 mm × 9.0 mm, was interpreted as most consistent with enlargement of the presumed splenic lymph node. Another rounded mass adjacent to the abdominal aorta, measuring 15.6 mm × 11.2 mm, was interpreted as most consistent with enlargement of the presumed lumboaortic lymph node.

No definite mass lesions or overt ultrasonographic evidence of clinically significant parenchymal involvement were detected within the abdominal organs. A solitary hyperechoic nodule measuring approximately 4 mm in diameter was observed within the left hepatic lobe. Additionally, a 4.5 mm hypoechoic nodule was identified in the splenic body. Both lesions were small, well-demarcated, and not associated with architectural distortion.

Whole-body contrast-enhanced computed tomography (CT) was performed using a 64-slice helical CT scanner (Optima CT660; GE HealthCare, Chicago, IL, USA) to evaluate systemic involvement. Intravenous contrast enhancement was achieved with iohexol (Omnipaque™ 300; GE HealthCare) administered at 600 mg iodine/kg using a power injector (MEDRAD^®^ Stellant; Bayer, Leverkusen, Germany). Because the clinically relevant abnormalities were concentrated in the thoracic and abdominal cavities, the findings are described below with emphasis on those regions.

The largest mass was again identified adjacent to the ileocolic junction and measured 55 mm × 45 mm × 35 mm (length × width × height). The lesion demonstrated heterogeneous contrast enhancement, with multiple enhancing regions interspersed with minimally enhancing hypoattenuating areas (Figure 1B). On CT, the jejunal and colic lymph nodes could not be clearly distinguished separately from this mass; therefore, the lesion was interpreted as most consistent with a markedly enlarged presumed abdominal lymph node. Although the mass was closely apposed to the ileum and colon, no definitive luminal continuity or mural origin was identified. An additional smaller mass measuring 10.2 mm was present adjacent to the primary lesion and demonstrated heterogeneous contrast enhancement, appearing relatively hypoattenuating compared with the larger mass.

A mass adjacent to the splenic vein, measuring 15.8 mm × 13.4 mm × 14.2 mm, was interpreted as most consistent with enlargement of the presumed splenic lymph node and showed heterogeneous contrast enhancement (Figure 1D). A second mass located to the left of the abdominal aorta, measuring 15.0 mm × 11.2 mm × 12.9 mm, was interpreted as most consistent with enlargement of the presumed lumboaortic lymph node and similarly demonstrated heterogeneous enhancement (Figure 1E). Within the left hepatic lobe, a small hyperattenuating nodule was observed. No additional focal hepatic lesions were identified. The spleen did not demonstrate definitive focal abnormalities on CT.

Additional soft tissue masses were identified at expected thoracic lymph node stations. The sternal lesion measured 16.6 mm × 13.8 mm × 11.3 mm. Multiple cranial mediastinal lesions were also present, each exceeding 5 mm in thickness; the largest measured 18.6 mm × 15.5 mm × 14.7 mm (Figure 1F). No pulmonary nodules, pleural effusion, or other thoracic abnormalities were detected. No osteolytic lesions or osseous abnormalities were identified on whole-body CT.

Based on the presence of multicentric masses located predominantly at expected lymph node stations, without identification of a primary visceral mass, together with the patient’s advanced age and systemic distribution of lesions, a neoplastic process was considered the leading imaging differential diagnosis, with lymphoma considered most likely. Non-neoplastic causes of multicentric lymph node enlargement, including feline infectious peritonitis and systemic infectious diseases of viral, bacterial, or fungal origin, were also considered.

Ultrasound-guided fine-needle aspiration was performed on the largest mass adjacent to the ileocolic junction and on a nearby nodule. Cytologic evaluation of both lesions yielded highly cellular samples predominantly composed of round cells (Figure 1C). The neoplastic cells exhibited eccentrically positioned nuclei with coarse chromatin and occasional prominent nucleoli. A subset of cells demonstrated pale perinuclear clearing consistent with a perinuclear halo. Binucleated and occasional multinucleated forms were observed, and moderate anisocytosis and anisokaryosis were present. These cytomorphologic features were compatible with plasmacytic differentiation.

Because no osteolytic lesions or other imaging evidence of primary osseous involvement were identified on whole-body CT, and because the lesions were distributed predominantly at expected lymph node stations, the overall clinical, cytologic, and imaging findings supported a multicentric round cell neoplasia with plasmacytic differentiation. Plasma cell neoplasia remained an important diagnostic consideration; however, plasmacytic lymphoma also remained an important differential diagnosis, and definitive classification was not possible. Further immunophenotypic characterization was therefore pursued. Flow cytometric analysis of the aspirated mass identified a predominantly small-sized cell population lacking expression of mature B-cell (CD21) and T-cell (CD4, CD8, CD5) markers. Although an immunophenotype consistent with conventional B- or T-cell lymphoma was not demonstrated, these findings were not specific for plasma cell lineage. In conjunction with the cytomorphologic features suggestive of plasmacytic differentiation, the flow cytometric findings were interpreted only as indirect, non-specific supportive evidence. Histopathologic examination with immunohistochemical characterization, bone marrow evaluation, and assessment for monoclonal gammopathy were recommended to further refine disease classification and exclude occult multiple myeloma; however, these procedures were declined by the owner.

Eight days after the initial diagnosis, the owner elected to proceed with radiation therapy in combination with systemic chemotherapy. Surgical excision was declined. A planning CT examination was performed for radiation treatment planning. The patient was positioned in ventral recumbency using a custom-made vacuum immobilization cushion (Chunsung, Seoul, Republic of Korea) to ensure reproducible setup.

The gross tumor volume (GTV) was defined as the contrast-enhancing portion of the mass identified on CT. The planning target volume (PTV) was generated by applying a 5 mm isotropic expansion to the GTV to account for setup uncertainties and potential organ motion. Treatment was delivered using a 6-MV linear accelerator equipped with 5 mm multileaf collimators (Clinac iX; Varian Medical Systems, Palo Alto, CA, USA). Two full-arc coplanar volumetric modulated arc therapy (VMAT) beams were utilized, and inverse treatment planning was performed using Eclipse™ software version 13.7.33 (Varian Medical Systems).

A total dose of 36 Gy was prescribed to the PTV, planned as six fractions of 6 Gy each administered on a weekly schedule. Treatment planning ensured that at least 95% of the PTV and 99% of the GTV received 100% of the prescribed dose. Organs at risk, including the colon, small intestine, kidneys, ureters, liver, and spinal cord, were contoured, and established dose constraints were applied during plan optimization to minimize normal tissue exposure. Temporary treatment interruptions occurred following the third fraction due to pneumonia and again between the fifth and sixth fractions secondary to recurrent respiratory complications and declining clinical condition. Concurrent systemic chemotherapy was initiated with prednisolone (0.3 mg/kg orally twice daily) and melphalan (0.125 mg/kg orally).

Daily cone-beam CT (CBCT) was acquired prior to each fraction to verify patient positioning and reproduce the treatment setup established during planning. Following completion of the third fraction, interval reduction in tumor size was subjectively noted on serial CBCT imaging. Accordingly, a repeat contrast-enhanced CT examination was performed to formally assess treatment response and facilitate adaptive replanning.

**Figure 2:**
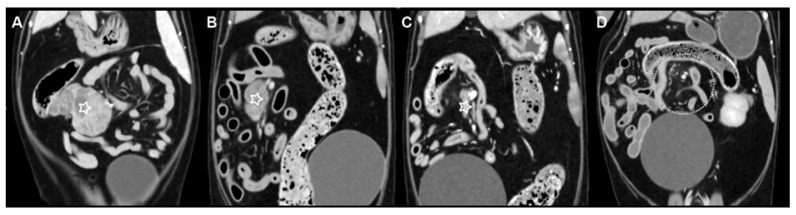
Serial contrast-enhanced computed tomography (CT) images (dorsal plane) demonstrating treatment response of the mass located adjacent to the ileocolic junction following hypofractionated radiotherapy (window level, 40; window width, 400). For all panels, the right side of each image corresponds to the patient’s left. (**A**) Pre-treatment CT showing the contrast-enhancing mass (white star), with a gross tumor volume (GTV) of 42.5 cm^3^. (**B**) CT obtained after three fractions of radiotherapy demonstrating marked reduction in mass size (white star), with a GTV of 9.6 cm^3^. (**C**) CT obtained after completion of six fractions demonstrating near-complete regression of the irradiated mass (white star), with a residual GTV of 0.8 cm^3^. (**D**) Follow-up CT performed one month after completion of radiotherapy. The region corresponding to the previous tumor location (white dotted circle) contains no identifiable residual contrast-enhancing lesion, consistent with complete radiologic resolution.

On repeat CT, the mass adjacent to the ileocolic junction demonstrated a marked reduction in size. The GTV decreased from 42.5 cm^3^ at initial planning to 9.6 cm^3^ following three fractions of radiotherapy, representing an approximate 77% reduction in tumor volume (Figure 2A,B). In light of this substantial decrease in target volume, adaptive replanning was undertaken to account for the modified tumor geometry. No significant interval change in size was observed in the presumed lumboaortic or splenic lymph nodes. Similarly, the presumed sternal and cranial mediastinal lymph nodes did not show appreciable interval reduction.

Immediately following completion of the sixth fraction of radiotherapy, a contrast-enhanced CT examination was performed to evaluate treatment response. The mass adjacent to the ileocolic junction demonstrated marked interval reduction in size, measuring 23.0 mm × 6.7 mm × 11.5 mm. The GTV was reduced to 0.8 cm^3^, corresponding to an approximate 98% reduction relative to the initial pre-treatment volume of 42.5 cm^3^ (Figure 2C). In contrast, interval progression was observed in all non-irradiated presumed nodal lesions. The presumed splenic lymph node had enlarged to 20.2 mm × 18.3 mm × 16.9 mm. The presumed lumboaortic lymph node had enlarged to 19.9 mm × 14.5 mm × 13.8 mm. The presumed sternal lymph node had enlarged to 22.7 mm × 16.5 mm × 15.4 mm. Multiple presumed cranial mediastinal lymph nodes also demonstrated interval enlargement, with the largest measuring 19.3 mm × 15.2 mm × 16.2 mm. Given the progression of non-irradiated presumed nodal lesions, the dosage of melphalan was increased to 0.15 mg/kg orally once daily.

**Figure 3:**
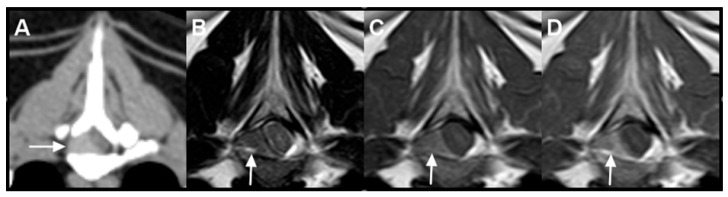
Cross-sectional imaging of the extradural mass identified at the level of the T5 vertebra. For all panels, the right side of each image corresponds to the patient’s left. (**A**) Post-contrast computed tomography (CT), axial plane (window level, 40; window width, 400), demonstrating a contrast-enhancing extradural mass within the right aspect of the vertebral canal (white arrow). (**B**) T2-weighted magnetic resonance imaging (MRI), axial plane, showing the lesion to be iso- to mildly hypointense relative to the spinal cord gray matter (white arrow). (**C**) T1-weighted MRI, axial plane, in which the mass appears hyperintense relative to the spinal cord gray matter (white arrow). (**D**) Post-contrast T1-weighted MRI, axial plane, demonstrating heterogeneous enhancement of the extradural mass (white arrow).

One month after completion of radiotherapy, the patient was re-presented for acute-onset pelvic limb weakness. Contrast-enhanced CT was performed for restaging. No residual contrast-enhancing lesion was identified at the site of the previously irradiated mass adjacent to the ileocolic junction, consistent with complete radiologic resolution (Figure 2D). The presumed splenic lymph node had decreased in size to 14.4 mm × 11.0 mm × 12.0 mm. However, interval progression was observed in the other presumed nodal lesions. The presumed lumboaortic lymph node had enlarged to 20.0 mm × 18.7 mm × 17.6 mm, and the presumed sternal lymph node had further enlarged to 29.7 mm × 24.3 mm × 29.2 mm. All presumed cranial mediastinal lymph nodes demonstrated continued enlargement, with the largest measuring 19.1 mm × 17.2 mm × 18.1 mm. Additionally, a newly developed contrast-enhancing mass was identified at the level of the T5 vertebra within the right dorsolateral aspect of the vertebral canal (Figure 3A). No associated osteolysis was detected.

Magnetic resonance imaging (MRI) was subsequently performed for further characterization of the spinal lesion. At the T5 level, an extradural mass was identified along the right aspect of the vertebral canal, measuring approximately 11.8 mm × 5.55 mm × 3.2 mm. The lesion was iso- to mildly hypointense relative to spinal cord gray matter on T2-weighted images and hyperintense on T1-weighted images (Figure 3B,C). Following contrast administration, the mass demonstrated heterogeneous enhancement (Figure 3D). The lesion resulted in leftward displacement and compression of the spinal cord; however, no intramedullary signal abnormalities were observed. No vertebral marrow signal alteration or cortical bone lysis was identified.

In the context of concurrent progression of multiple presumed nodal lesions, the newly identified T5 extradural spinal lesion was considered suspicious for additional neoplastic involvement. However, its etiology could not be definitively determined on imaging alone, and cytologic or histopathologic confirmation was not obtained. In light of suspected systemic progression, cyclophosphamide was incorporated into the chemotherapeutic regimen at a dose of 50 mg/m^2^.

Given the progression of presumed thoracic nodal lesions and the development of the T5 extradural mass, palliative-intent radiotherapy was planned for these sites. A total dose of 36 Gy, delivered in six fractions of 6 Gy each, was prescribed. Two fractions were administered; however, the owner elected to discontinue further treatment and declined additional diagnostic evaluation. The patient was subsequently lost to follow-up, and long-term outcome could not be assessed. The final clinical contact occurred 114 days after initiation of the first course of radiotherapy directed at the abdominal mass.

## 3. Discussion

This report describes a multicentric round cell neoplasia with plasmacytic differentiation in a cat, documented with comprehensive multimodal cross-sectional imaging. This case should be interpreted in light of important diagnostic limitations, including the absence of histopathology, immunohistochemistry, bone marrow evaluation, and assessment for monoclonal gammopathy. Accordingly, although extramedullary plasmacytoma remained within the differential clinicopathologic interpretations, definitive classification was not possible. In the present case, multimodal imaging facilitated initial staging and objective longitudinal assessment of tumor burden. Serial imaging demonstrated marked regression of the irradiated mass adjacent to the ileocolic junction following radiotherapy, whereas non-irradiated presumed nodal lesions showed progressive enlargement despite concurrent chemotherapy. Collectively, these findings highlight the value of longitudinal imaging in delineating disease distribution and documenting differential treatment response in feline round cell neoplasia with plasmacytic differentiation.

At initial presentation, cross-sectional imaging revealed multicentric masses distributed predominantly at expected abdominal and thoracic lymph node stations, without identification of a primary visceral mass. The systemic distribution of these presumed nodal lesions and the advanced age of the patient favored lymphoma as the leading imaging differential diagnosis, as lymphoma is the most common cause of generalized lymph node enlargement in cats [18,19]. However, cytologic evaluation demonstrated plasmacytic differentiation, illustrating that round cell neoplasia with plasmacytic differentiation may closely mimic lymphoma on imaging studies. Although uncommon, neoplasia with plasmacytic differentiation should therefore be included in the differential diagnosis when multicentric presumed nodal enlargement is identified in feline patients. This case underscores the importance of integrating imaging findings with cytologic assessment to improve diagnostic classification in cases of generalized lymph node enlargement.

Definitive classification of extramedullary plasmacytoma typically requires histopathologic evaluation with immunohistochemical confirmation [20]. In the present case, histologic assessment, bone marrow evaluation, and additional diagnostic testing were recommended but declined by the owner. Therefore, the available findings support a multicentric round cell neoplasia with plasmacytic differentiation, with plasma cell neoplasia remaining an important diagnostic consideration. Flow cytometric analysis did not demonstrate expression of mature B-cell (CD21) or T-cell (CD4, CD8, CD5) markers, thereby decreasing the likelihood of a conventional B- or T-cell lymphoma immunophenotype. Nevertheless, terminally differentiated plasma cells may lack typical surface B-cell markers, and immunophenotyping alone cannot conclusively establish plasma cell lineage. Considering the multifocal distribution of lesions, extramedullary plasmacytoma remained among the differential clinicopathologic interpretations; however, definitive classification was limited by the absence of histopathology, immunohistochemistry, and bone marrow evaluation.

Multiple myeloma was also considered in the differential diagnosis. Commonly accepted diagnostic criteria for feline multiple myeloma include osteolytic bone lesions, monoclonal gammopathy, hypercalcemia, and bone marrow plasmacytosis [16,17,21]. In this patient, osteolysis was not identified on cross-sectional imaging, hypercalcemia was absent, and Bence–Jones proteinuria was not detected. Monoclonal gammopathy could not be assessed because serum protein electrophoresis was not performed. While bone marrow evaluation was not undertaken and multiple myeloma therefore could not be definitively excluded, the absence of characteristic systemic features together with the predominance of presumed nodal soft tissue involvement made overt multiple myeloma less likely.

Plasma cell neoplasms are generally regarded as radiosensitive tumors, and radiotherapy is considered an effective modality for achieving local control [20]. In human medicine, solitary plasmacytoma of bone (SPB) and solitary extramedullary plasmacytoma (SEP) are primarily managed with definitive radiotherapy, typically administered at total doses ranging from 35 to 50 Gy in conventional daily fractions of 1.8–2 Gy [22,23]. Lesions measuring less than 5 cm are often controlled with 35–40 Gy, whereas larger or bulky tumors may receive 45–50 Gy [22,23]. Reported local control rates in retrospective series commonly range from 80% to nearly 100% [22,23]. In contrast, lower hypofractionated doses (8–30 Gy) are frequently employed for palliative management of multiple myeloma-associated osseous lesions, providing high rates of symptomatic relief and functional improvement [22].

Veterinary data are comparatively limited but similarly support the radiosensitivity of plasma cell tumors. In a retrospective study of 30 dogs with macroscopic plasma cell tumors, both definitive-intent protocols (median 48 Gy delivered in 3–4.2 Gy fractions) and palliative-intent regimens (median 30 Gy delivered in 4–10 Gy fractions) achieved high overall response rates (95% complete or partial response), with durable local control and prolonged progression-free survival [24]. Reports in cats remain scarce; however, hypofractionated radiotherapy (e.g., 12 Gy delivered as 4 Gy × 3 fractions) has resulted in long-term local control in solitary osseous plasmacytoma [25].

In the present case, a hypofractionated protocol (36 Gy delivered in six fractions of 6 Gy) was selected to balance anticipated radiosensitivity with clinical and logistical considerations. Serial imaging demonstrated rapid and substantial volumetric reduction in the irradiated mass located adjacent to the ileocolic junction, culminating in complete radiologic resolution at short-term follow-up. This marked local response is consistent with the recognized radiosensitivity of plasma cell neoplasia and further supports the utility of radiotherapy as an effective modality for local disease control in this case. In contrast, progressive enlargement of non-irradiated presumed nodal lesions occurred concurrently, illustrating the dissociation between successful local tumor control and ongoing systemic disease evolution.

Several limitations should be considered when interpreting this case. Histopathologic examination with immunohistochemical confirmation was recommended but declined by the owner; consequently, the diagnosis was based on cytomorphologic features consistent with plasmacytic differentiation in conjunction with supportive but non-specific flow cytometric findings. Although the cytologic morphology supported plasmacytic differentiation and immunophenotyping did not demonstrate an immunophenotype consistent with conventional B- or T-cell lymphoma, cytologic sampling did not permit evaluation of tissue architecture, including confirmation of nodal architecture, assessment of architectural effacement, or characterization of the pattern of tissue infiltration. In addition, the absence of immunohistochemical characterization precluded definitive histologic classification.

In addition, bone marrow evaluation was not performed; therefore, occult multiple myeloma cannot be completely excluded. Progressive enlargement of non-irradiated presumed nodal lesions and the subsequent development of a T5 extradural spinal mass were considered suspicious for additional disease involvement in the context of systemic progression; however, cytologic or histologic confirmation of these lesions was not obtained. MRI classification of the spinal lesion was also limited by overlap among round cell neoplasms. In feline spinal lymphoma, MRI findings are heterogeneous and may include epidural soft tissue-centered lesions, paravertebral masses with vertebral canal invasion, or nerve root-centered disease, with or without vertebral involvement, and lesions are most commonly T1-isointense [26]. By comparison, plasma cell tumor/multiple myeloma more often demonstrate a bone-centered pattern with vertebral marrow involvement and associated extradural material, and T1 hyperintensity has been reported more commonly in plasma cell tumor/multiple myeloma than in lymphoma [27,28]. Notably, the T1 hyperintensity of the T5 extradural lesion was more compatible with previously reported plasma cell tumor/multiple myeloma MRI patterns, whereas feline spinal lymphoma is more commonly T1-isointense. Nevertheless, overlap exists, and this feature alone was insufficient to establish lesion lineage [26,27,28]. Accordingly, the spinal lesion in the present case remained presumptive despite suspicion for additional neoplastic involvement. As an individual case report, these observations should be interpreted cautiously, yet they provide clinically relevant insight into the imaging manifestations, treatment response, and potential patterns of progression of feline round cell neoplasia with plasmacytic differentiation.

In summary, this report describes a multicentric round cell neoplasia with plasmacytic differentiation in a cat, with detailed imaging documentation of both therapeutic response and subsequent systemic progression. This case adds to the limited literature on feline round cell neoplasia with plasmacytic differentiation and nodal-predominant involvement and supports inclusion of neoplasia with plasmacytic differentiation among the differential diagnoses for multicentric presumed nodal enlargement. Serial multimodal imaging was essential for delineating disease distribution, objectively quantifying treatment response, and identifying progressive lesions over time. Although radiotherapy achieved complete radiologic resolution of the mass adjacent to the ileocolic junction, concurrent progression at distant sites underscored the dissociation between effective local tumor control and ongoing systemic disease evolution. Together, these findings contribute to a more comprehensive understanding of the imaging features, treatment responsiveness, and clinical behavior of feline round cell neoplasia with plasmacytic differentiation, and may aid in guiding diagnostic evaluation and therapeutic decision-making in similar cases.

## 4. Conclusions

In conclusion, this report describes a multicentric round cell neoplasia with plasmacytic differentiation in a cat, with detailed serial multimodal imaging documentation of treatment response and subsequent systemic progression. The irradiated mass adjacent to the ileocolic junction exhibited rapid volumetric regression followed by complete radiologic resolution, whereas non-irradiated presumed nodal lesions progressed over time, highlighting the dissociation between successful local tumor control and ongoing systemic disease evolution. Although definitive classification was limited by the absence of histopathology, immunohistochemistry, bone marrow evaluation, and assessment for monoclonal gammopathy, this case contributes to the current understanding of the imaging spectrum, clinical behavior, and treatment responsiveness of feline round cell neoplasia with plasmacytic differentiation, and underscores the importance of longitudinal imaging in therapeutic assessment and clinical management.

## Data Availability

The original contributions presented in this study are included in the article. Further inquiries can be directed to the corresponding author.
